# Transgenic expression of IL-7 regulates CAR-T cell metabolism and enhances in vivo persistence against tumor cells

**DOI:** 10.1038/s41598-022-16616-2

**Published:** 2022-07-22

**Authors:** Li Li, Qing Li, Zi-Xun Yan, Ling-Shuang Sheng, Di Fu, Pengpeng Xu, Li Wang, Wei-Li Zhao

**Affiliations:** 1 State Key Laboratory of Medical Genomics, National Research Center for Translational Medicine at Shanghai, Shanghai Institute of Hematology, Ruijin Hospital, Shanghai Jiao Tong University School of Medicine, 197 Rui Jin Er Road, Shanghai, 200025 China; 2 Shanghai Centre for Clinical Laboratory, Shanghai, China

**Keywords:** Immunology, Adaptive immunity

## Abstract

Chimeric antigen receptor (CAR) T-cell therapy has emerged as a promising novel therapeutic approach. However, primary and secondary resistance to CAR-T cell therapy is commonly encountered in various clinical trials. Despite the comprehensive studies to elucidate the mechanisms of resistance, effective resolution in clinical practice is still elusive. Inadequate persistence and subsequent loss of infused CAR-T cells are proposed major resistance mechanism associated with CAR-T cell treatment failure. Thus, we generated CAR-T cells armored with IL-7 to prolong the persistence of infused T-cells, particularly CD4 + T cells, and enhanced anti-tumor response. IL-7 increased CAR-T-cell persistence in vivo and contributed to the distinct T-cell cytotoxicity profile. Using mass cytometry (CyTOF), we further assessed the phenotypic and metabolic profiles of IL-7-secreting CAR-T cells, along with conventional CAR-T cells at the single-cell level. With in-depth analysis, we found that IL-7 maintained CAR-T cells in a less differentiated T-cell state, regulated distinct metabolic activity, and prevented CAR-T-cell exhaustion, which could be essential for CAR-T cells to maintain their metabolic fitness and anti-tumor response. Our findings thus provided clinical rationale to exploit IL-7 signaling for modulation and metabolic reprogramming of T-cell function to enhance CAR-T cell persistence and induce durable remission upon CAR-T cell therapy.

## Introduction

Since chimeric antigen receptor (CAR) T-cell therapy has gained a tremendous clinical success, FDA approved four CAR-T cell products for treating hematologic malignancies^1^. CAR-T cell clinical trials have shown durable remissions in patients with relapsed or refractory diseases, including non-Hodgkin lymphoma^2^, multiple myeloma^3^, acute lymphoblastic leukemia (ALL)^4^, and chronic lymphocytic leukemia^5^. Although the clinical responses are encouraging, 30–50% of patients could experience relapse after achieving complete remission in long term follow-up^4,6^. Most studies on resistance mechanisms of CAR-T cell therapy point to antigen loss, CAR-T cell dysfunction and lack of CAR-T cell persistence^7^. The improvement of CAR structure design is necessary to overcome these limitations to maximize the clinical benefits, by optimizing antigen recognition and binding domains^8–10^, co-stimulation domains^11,12^, overcoming antigen escape by targeting multiple antigens^13,14^, and armoring CAR with constitutively expressed cytokines^15–17^.

Although previous studies showed that IL-7 enhances CAR-T cell proliferation in the setting of CD19-CAR-ζ that contains CD19-binding domain fused to CD8 hinge and transmembrane domains, it was known as the first-generation CAR^18^. It has been well-established that signaling mediated by CD3ζ alone was insufficient to induce T cell response and led to failure in T cell persistence and activity *in vivo*^19^. Moreover, the co-stimulatory domain was demonstrated to play an important role in CAR-T cell persistence and proliferation. FDA-approved CAR-T products contained either CD28 or 4-1BB co-stimulatory domains, are associated with high response rates. The evolution witnessed in CAR design encourages us to investigate the utility of a novel CAR-T cell product with combination of co-stimulatory CD28 signaling domain and secreted IL-7 in CAR transgene.

In this study, we chose to generate CAR-T cells to target the most commonly targeted antigen, CD19, to test the proof of concept that CAR-T cells with CD28 co-stimulatory domain and secreting IL-7 demonstrate improved cytotoxicity and superior persistence. It was shown that T cells require to have an efficient metabolic capacity to generate a long-lasting energy supply for a more effective immune response^20^ and activate different metabolic pathways based on their differentiation and memory status^21^. Thus, we employed mass cytometry (CyTOF) to characterize the metabolic profiles and phenotypic traits of IL-7 secreting CD19 CAR T-cells with CD28 co-stimulatory domain at the single-cell level and examine the impact of IL-7 addition to CAR construct on anti-tumor response of CAR-T cells in vitro and in vivo.

## Results

### IL-7-secreting CAR-T cells with CD28 co-stimulation demonstrate comparable cytotoxic response

**Figure 1:**
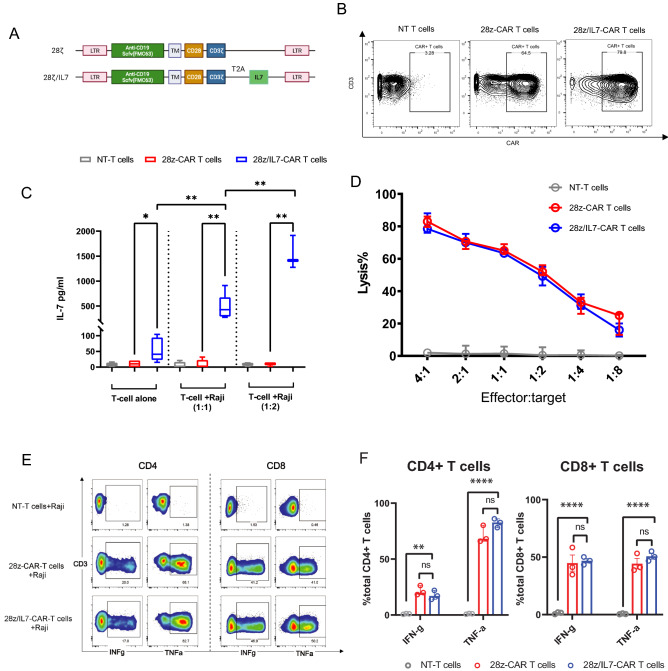
IL-7-secreting CAR-T cells with CD28 co-stimulation demonstrate comparable cytotoxic response. (**A**) A schematic illustration of the vectors used to transduce T cells. (**B**) Flow plots show the CAR expression in non-transduced (NT) T cells, 28z-CAR-T cells, and 28z/IL-7-CAR-T cells, the expression of CAR was detected using anti-human IgG antibody . (**C**) Box plot shows the IL-7 concentration of NT T cells, 28z-CAR-T cells, and 28z/IL-7-CAR-T cells in three different culture conditions : T-cell alone, T-cell cultured with Raji cells at 1:1 ratio, and T-cell cultured with Raji cells at 1:2 ratio. The color indicates different gene-engineered T cells. (*p < 0.05, **p < 0.01, n = 3, unpaired *t* test, Median ± SEM) (**D**) Bar plot shows the percentage of Raji cells (target) lysed by NT T cells (grey), 28z-CAR-T cells (red), and 28z/IL-7-CAR-T cells (blue) as measured by BLI based cytotoxicity assay for 18 h. (**E**) Plots Representative plots of cytokine production of NT T cells, 28z-CAR-T cells, and 28z-CAR-T cells against Raji cells. (**F)** Bar plots show the statistics analysis of cytokine production from 3 independent experiments (median with interquartile range) (**p < 0.01, ****p < 0.0001, n = 3, unpaired t test).

IL-7 is known to enhance the proliferation and survival of T cells^22^. Previous studies revealed that IL-7 promoted the anti-tumor efficacy of first generation CAR-T cells (CD3ζ only), but not cell persistence *in vivo*^18^. Since the second generation of CAR-T cells (CD28/4-1BB co-stimulatory with CD3ζ) are broadly utilized in clinical trials, we sought to investigate the in vitro and in vivo efficacy of a novel CAR construct that contains a transgene encoding secretable IL-7 and CAR molecule with CD28 co-stimulatory domain. We generated anti-CD19-CAR T cells using a lentiviral vector encoding anti-CD19 Scfv (FMC63), CD28 plus CD3ζ as intracellular signaling domains and/or IL-7 (Fig. 1A). Per previously approaches^17^, the gene of CD19-CAR with IL-7 was transduced to human primary T cells efficiently and we achieved high transduction rates (> 70%) (Fig. 1B). To ascertain that IL-7 was produced in CAR-T cells, we then tested levels of IL-7 in the supernatant of non-transduced (NT) T cells, 28z-CAR-T cells and 28z/IL-7-CAR-T cells. Notably, IL-7 was detected in the supernatant of 28z/IL-7-CAR-T cells cultured alone overnight but neither in NT T cells nor 28z-CAR-T cells (Fig. 1C). As known, IL-7 is produced by stomal cells and fibroblastic reticular cells^23^ but not by primary T-cells. Thus, the detected IL-7 in the supernatant was produced by 28z/IL-7-CAR-T cells having the transgene encoding IL-7. This confirms that the transgene integrated into T-cell genome is efficiently transcribed and translated, leading to production and secretion of IL-7 by transduced T cells. To evaluate the correlation between IL-7 production and antigen-dependent CAR-T cell activation, we also quantified IL-7 level in the supernatants after co-culturing CAR-T cells with Raji cells at 1:1 and 1:2 ratios overnight. Indeed, the concentration of IL-7 was significantly higher in the supernatant of T cells cocultured with Raji cells, particularly, 28z/IL-7-CAR-T cells produced even higher IL-7 at 1:2 effector to target (E:T) ratio, while we didn’t observe IL-7 in any supernatant of NT-T cells and 28z-CAR-T cells either in the absence or presence of target cells(Fig. 1C), indicating primary T-cells were successfully genetically engineered to produce IL-7 for maintaining T-cell fitness and persistence, and express CAR molecules to target CD19 expressing target cells. Importantly, CAR-T cell activation in an antigen dependent manner further drove IL-7 production. The boosted IL-7 levels in the presence of target cells is most probably due to activation of plethora of downstream pathways in response to CAR T cell signaling that could crosstalk with CAR promotor, thus leading to augment transcription of genome-integrated CAR transgene^24^. Next, we sought to assess the cytotoxicity against Raji cells. First, we measured the specific lysis of tumor cells after coculture with CAR-T cells at five different E:T ratios (4:1, 2:1, 1:1, 1:2, 1:4, and 1:8) using Bioluminescence Imaging(BLI)-based cytotoxicity assay (see methods). 28z/IL-7-CAR-T cells were as effective as conventional CAR-T cells (28z-CAR-T cells) to mediate tumor cell lysis (Fig. 1D). To investigate cytokine secretion profiles of 28z/IL-7-CAR-T cells against Raji cells, we measured the IFNγ and TNFα response after co-culturing with tumor cells using flow cytometry. In line with killing assay , the fractions of IFNγ and TNFα-producing CD4 + and CD8 + 28z/IL-7-CAR T cells upon co-culturing with Raji cells, were comparable to 28z-CAR-T cells and were significantly higher compared to NT-T cells (Fig. 1E,F). These findings indicate that addition of IL-7 does not alter cytokine expression profiles of CAR T-cells.

### IL-7 preserves a less differentiated CAR-T cell phenotype and mitigates exhaustion of CD4 + CAR-T cells

**Figure 2:**
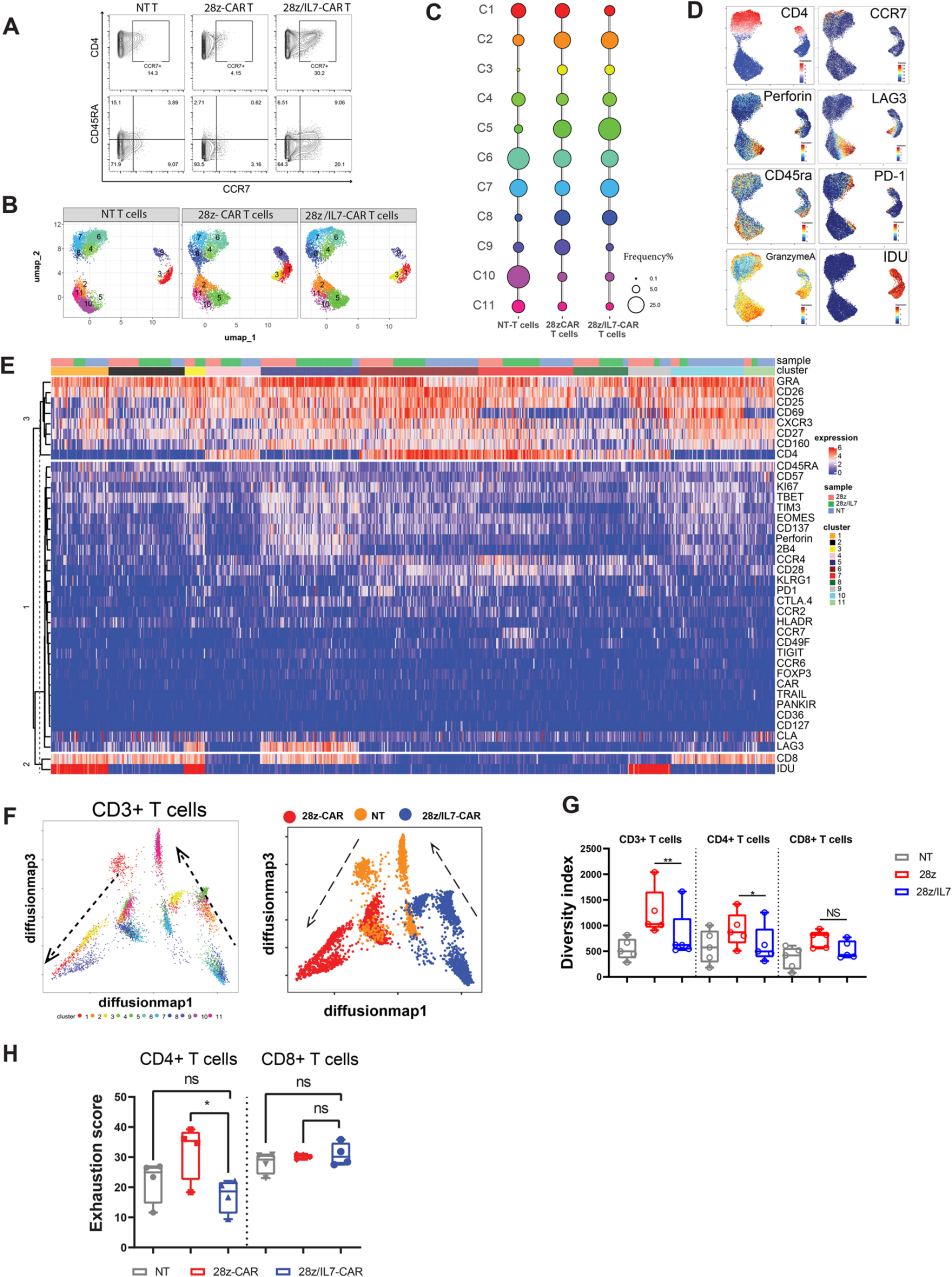
IL-7 preserves a less differentiated CAR T-cell phenotype and mitigates exhaustion of CD4 + CAR-T cells. (**A**) FACS plot shows the expression of CCR7 and CD45RA in NT T cells, 28z-CAR-T cells, and 28z/IL-7-CAR-T cells. (**B**) UMAP plots show the clusters distribution in three different samples (NT T cells, 28z-CAR-T cells, and 28z/IL-7-CAR-T cells). (**C**) Bubble plot shows the percentage of cluster 1 to 11 in NT T cells, 28z-CAR-T cells, and 28z/IL-7-CAR-T cells. (**D**) Expression of CD4, CCR7, perforin, LAG3, CD45RA, PD-1, Granzyme A, and IDU in UMAP analysis. (**E**) Heatmap shows the levels of the proteomic markers for 3 samples with 11 clusters. Each column represents a cell and each row indicates a marker. (**F**) Diffusion maps show trajectory analysis of NT T-cells, and 28z and 28z/IL-7 CAR-T cell and cells are colored per cluster. Right plot shows the localization of cells from three different samples (NT T cells, 28z-CAR-T cells, and 28z/IL-7-CAR-T cells) using diffusion map algorithm. The dashed line with arrow shows the trajectory direction according to the expression of T-cell differentiation markers (CCR7, CD45RA). (**G**) Bar plot shows the diversity index of CD3 + , CD4 + and CD8 + T cells in NT T cells, 28z-CAR-T cells, and 28z/IL-7-CAR-T cells. Diversity index was calculated by using 32 T cell related markers as described in the methods. Each dot represents one independent experiment (n = 4) (*p < 0.05, **p < 0.01,unpaired t test, Median ± SEM). (**H**) Bar plot shows the exhaustion score of CD4 + and CD8 + T cells in NT T cells, 28z-CAR-T cells, and 28z/IL-7-CAR-T cells, each dot represents one independent experiment (n = 4) (*p < 0.05, unpaired t test, Median ± SEM).

Next, we sought to investigate of the potential impact of IL-7 on CAR-T cell phenotypic profiles since IL-7 is implicated in generation of less-differentiated T-cells with stem-like T-phenotypes and inducing self-renewal^25^. To this end, we performed mass cytometry (CyTOF) by utilizing a 34-antibody panel to assess distinct surface and intracellular features (Supplemental Table 1). For comparative analysis, we utilized unsupervised high-dimensional analytic approaches to achieve a broad overview of CAR-T cell phenotypic landscape. IL-7 is required for T-cell development as well as for maintaining of native and central memory T cell pool in peripheral blood. Thus, we hypothesized enforced IL-7 expression could retain the cells in a less differentiated state, maintain stemness and trigger self-renewal, which could translate to improved persistence in vivo since IL-7 may maintain the native and memorial T-cell phenotype to achieve the feature of self-renew and stemness in T cells. To test this notion, we assessed the differentiation states of IL-7 secreting CAR-T cells (28z/IL-7 CAR) and conventional CAR T-cells, and compared the expression of CCR7 and CD45RA, the traditional markers commonly used to assess T-cell differentiation state. Indeed, CD4 + CAR-T cells expressing IL-7 had higher levels of CCR7 while CD4 + CAR-T cells that with CD28 co-stimulatory domain and lacking IL-7 had lower levels of CCR7 (Fig. 2A). However, we did not observe the same pattern on CD8 + T cells (Supplemental Fig. 1A). It is worth noting that CD8 + T-cells with central memory phenotype are comparably low in healthy individuals and naïve CD8 + T-cells tend to differentiate to CCR7(-) effector memory and terminally differentiated CD8 + T-cells upon activation and memory formation. Next, we assessed the fractions of naïve (CD45RA + CCR7 +), central memory (CD45RA-CCR7 +), effector memory (CD45RA-CCR7-), and terminally differentiated (CD45RA + CCR7-) T-cell fractions of CD4 + 28z/IL-7 and 28z CAR-T cells. ^26^. In agreement with our notion, we found that the higher frequencies of naïve and central memory subsets were preserved in 28z/IL-7-CAR T cells after activation, transduction and expansion(Fig. 2A), indicating that IL-7 expression contributes to maintenance of a less-differentiated T-cell state ^22,27^. To elucidate the differentiation state of naïve-like T-cell population in 28z/IL-7-CAR-T cells, we assessed CXCR3 expression and we found that CD45RA + CCR7 + T cells expressed CXCR3^28^ (Supplemental Fig. 1B), one of the core set markers that differentiate stem cell-like memory T-cells (Tscm) from naïve T-cells, suggesting that the phenotypic profiles of naïve-like T-cell population are similar to Tscm phenotypic features^25,29^. With single-cell proteomic analysis, we unbiasedly compared distinct profiles of 28z/IL-7 and 28z CAR-T cells. First, we performed UMAP dimension reduction to visualize the data in two dimensions and assess marker expression patterns across the overall proteomic landscape. Unsupervised clustering analysis to identify cell populations with similar phenotypes identified 11 T cell clusters. The most segregated and distinct clusters were clusters 1, 3 and 9, which were differed with regards to IDU positivity which marks T-cells in S-phase of cell cycle^30^. CD4 + T cells and CD8 + T cells were distinctly segregated (Fig. 2B). We next determined and compared the cluster composition among NT, 28z/IL-7 and 28z CAR T cells , and observed that cluster C2, C5 and C8 were mainly found in CAR-T cell group rather than NT-T cells. C2 and C5 were CD8 + T cells expressing LAG3, PD-1, Perforin, and granzyme A while C8 corresponded to CD4 + T cells expressing CD57 and lacking CD69 and CD25 expression (Fig. 2C–E, Supplemental Fig. 1C), indicating the CAR signaling differentially modulated CD4 + and CD8 + T-cell phenotypic profiles and contributed to CD8 + CAR-T cell exhaustion. This is in line with previous studies showing that tonic CAR signaling induces T-cell exhaustion^31^. Notably, C9 marked proliferating CD4 + T cells that expressed Ki67 and PD-1 and preferentially enriched in 28z-CAR-T cells compared to IL-7-secreting CAR-T cells (Fig. 2C). In contrast, cells in C7 expressed CCR7 and the frequency was higher in 28z/IL-7-CAR-T cells compared to 28z CAR-T cells. Meanwhile, cells in C5 were enriched CD8 + T-cells having a functional cytotoxic T cell profile with high levels of perforin, LAG3 and granzyme A. These findings demonstrated that CAR induced a unique proteomic profile shared between 28z/IL-7 and 28z CAR-T cells and ectopic expression of IL-7 further altered the proteomic landscape of CAR-T cells and maintained CD4 + CAR-T cells in a less differentiated state.

We then performed trajectory analysis to gain insights into differentiation states of NT T-cells, and 28z/IL-7 and 28z CAR-T cells. We projected all the analyzed cells on two dimensions using the diffusion map algorithm and inferred the pseudo-time for each single-cell per differentiation state based on the expression of well-established T-cell differentiation markers(Supplemental Fig. 2A,B). We plotted 11 T-cell clusters identified through unsupervised clustering (Fig. 2B) using diffusion map algorithm to infer the association between different T-cell subpopulations with regards to differentiation state. As expected , less differentiated clusters (C6, C7 and C8) (bottom right) were distinctly situated compared to more differentiated T-cell clusters (C1, C3 and C9) (bottom left) (Fig. 2F). Then, we inferred the differentiation trajectory based on marker expression levels across clusters and deciphered distinct differentiation hierarchies. Interestingly, we observed 28z/IL-7-CAR-T cells were situated at earlier stage of differentiation while 28z-CAR-T cells were more differentiated (Fig. 2F). NT T-cells were positioned between 28z/IL-7 and 28z T-cells. There findings indicate that the IL-7 maintains CAR-T cells in a less differentiated state than CAR-T cells lacking IL-7. These findings support the notion that IL-7 may modulate tonic CAR signaling which drives T-cell differentiation and induces exhaustion since we observed that a prominent fraction of naïve-like and central memory CD4 + T-cells in 28z/IL-7 group (Supplemental Fig. 2C). Constitutively active signaling pathways could diversify T-cell phenotypic landscape and drive the emergence of multitude of distinct phenotypic subsets. Various diversity indices are utilized to assess the abundance, evenness and distribution of distinct T or NK-cell differentiation states^32,33^. We reasoned that relatively more active tonic signaling in 28z CAR-T cells could further induce differentiation, leading to emergence of a variety of differentiated T-cell subsets in comparison to 28z/IL-7 CAR-T cells. Thus, we assumed that the 28z CAR-T cells had higher diversity and measured the diversity index for each group. Of note, the diversity of CD3 + and CD4 + 28z-CAR-T cells was significantly higher than 28z/IL-7-CAR-T cells (Fig. 2G), revealing IL-7 shaped phenotypic profiles of CAR-T cells, particularly those of CD4 + T cells and limited diversification of T-cell compartment by mitigating T-cell differentiation. This could be particularly important for adoptive cell therapy since less differentiated T-cells show superior persistence. Multiple studies showed that tonic signaling from CAR induces T cells exhaustion ^31,34^. Therefore, we examined the exhaustion status of CAR-T cells and measured the exhaustion score for each group (See Methods). Interestingly, the exhaustion score based on the expression level of PD-1, CTLA-4, TIM-3 and LAG3 was higher in 28z CD4 + CAR-T cells compared to 28z/IL-7 CD4 + CAR-T cells, which indicates that the secretion of IL-7 may mitigate exhaustion secondary to tonic signaling in CD4 + CAR-T cells (Fig. 2H).

### IL-7 modulates the metabolic pathways in CAR-T cells upon antigen challenge

**Figure3:**
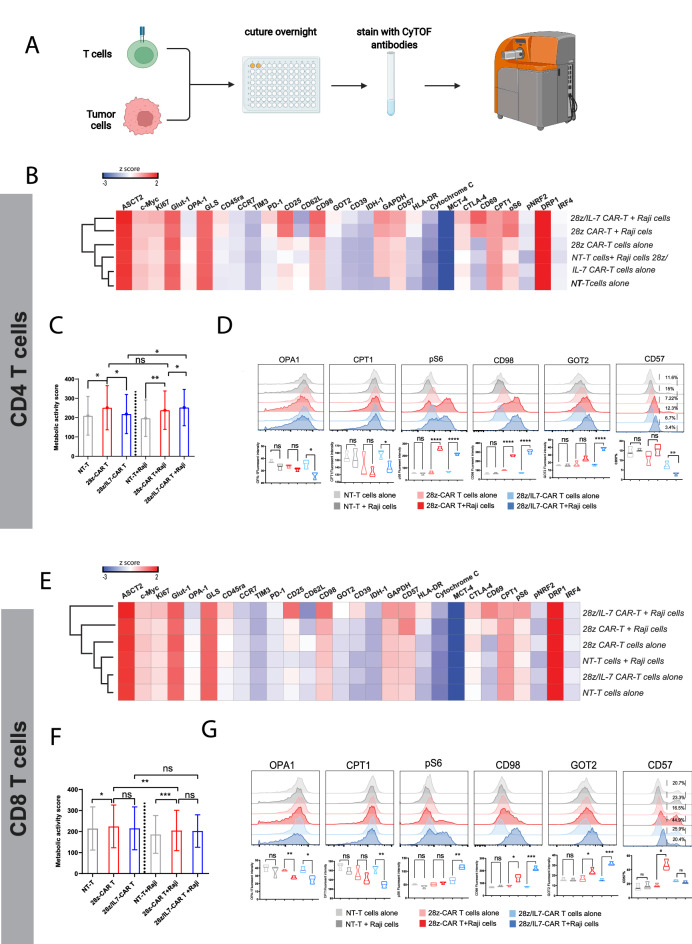
**IL-7 **modulates the metabolic pathways in CAR-T cells upon antigen challenge. (**A**) A schematic illustration of the experiment to assess metabolic activity. Heatmaps show the expression of each proteomic marker from CD4 + (**B**) and CD8 + (**E**) NT T cells, 28z-CAR-T cells, and 28z/IL-7-CAR-T cells in different culture conditions. Bar plots show the metabolic activity score calculated by the mean of expression of 17 markers assessed to metabolic pathways in CD4 + (**C**) and CD8 + (**F**) T cells, (Error bars indicate the Mean ± SEM, *p < 0.05, **p < 0.01, paired t test). Histogram plots show the expression level of OPA1, CPT1, pS6, CD98, GOT2 and CD57 and the bar plots indicate the median of expression from three independent experiments (n = 3, *p < 0.05, **p < 0.01,****p < 0.0001, unpaired *t* test, median ± SEM) in CD4 +** (D**) and CD8 + (**G**) T cells.

Distinct T cell activation states require metabolic programs compatible with their functional demands, and IL-7 is shown to regulate CAR-T cell differentiation states. Thus, we sought to investigate adaptive changes in metabolic profiles of CAR-engineered human primary T cells when challenged with tumor cells. To achieve an unbiased comparison among different CAR-T cells, we designed an experiment where we co-cultured NT T-cells, 28z-CAR-T cells, and 28z/IL-7-CAR-T cells with Raji cells at 1:1 ratio without cytokine support overnight and T-cells cultured alone were utilized as control for comparative analysis. Then the cells were stained with a collection of CyTOF antibodies using a metabolism-focused panel (supplemental Table S2) and data was generated on Mass Cytometer (Fig. 3A). We first analyzed the expression level of each marker using heatmaps and clustered the samples hierarchically to identify samples with similar metabolic profiles. Interestingly, the both resting CD4 + and CD8 + 28z/IL-7-CAR-T cells that cultured alone were computationally clustered with NT-T cells, which showed lower level of metabolic activity (Fig. 3B,E). To achieve a more simplified overview of the overall metabolic profiles of NT T cells and CAR-T cells in different culture conditions, we generated a metabolic score based on expression of 17 markers used to assess metabolic pathways(See Methods). Noteworthy, the resting IL-7 secreting CAR-T cells (CD4 +) had significantly decreased level of metabolic activity, as compared to NT-T cells and conventional CAR-T cells (Fig. 3C,F), indicating that IL-7 modulated CD4 + CAR-T cell metabolic activity and retained CD4 + CAR-T cells in a low level of metabolic activity in the resting stage. Interestingly, only IL-7-secreting CD4 + CAR-T cells rapidly increased their metabolic activity when co-cultured with Raji cells (Fig. 3C,F). Additionally, we observed that, unlike conventional CD4 + CAR-T cells (28z-CAR), the metabolic activity of conventional CD8 + CAR-T cells surprisingly decreased with tumor stimulation (Fig. 3F).

To gain insights into the mechanism of metabolic profile changes, we analyzed the markers individually. When Comparing the CAR-T cells in the presence or absence of tumor cells, we noticed that OPA1 and CPT1 were the only two markers downregulated in 28z/IL-7-CAR-T cells in the presence of tumor cells (Fig. 3D, G). As previous reported^35,36^, OPA-1 was required for memory T cells development, but not for effector T cells, and OPA1 deficiency induced stronger TCR signaling. Thus, we concluded that 28z/IL-7-CAR T cells acquired effector T cell functions by downregulating OPA-1 for eliminating tumor cells. These findings are similar to the pattern that we observed for CPT1, a marker related to fatty acid metabolism, since fatty acid (FA) metabolism is more critical for activation and rapid proliferation of memory CD4 + T cells^37^ rather than effector CD4 + T cells. We observed that naive and central memory CD4 + T cells expressed higher level of CPT1 and OPA1 than effector CD4 + T cells (Supplemental Fig. 3). Therefore, the effector T cells were active and required the adaptive metabolic activation against tumor cells. On the other hand, pS6 and CD98 were significantly higher increased in both conventional and IL-7 secreting CD4 + CAR-T cells co-cultured with tumor cells (comparably higher level of upregulation in 28z/IL-7 group) while GOT2, that plays a role in amino acid metabolism, was significantly upregulated in IL-7 secreting CD4 + CAR-T cells. (Fig. 3D). These findings suggest that IL-7 regulated the metabolic fitness of T-cells that enables their rapid adaptation and upregulation of a variety of metabolic pathways when challenged with tumor cells. Contrastingly, different from CD4 + CAR-T cells, the conventional CD8 + CAR-T cells slightly increased the expression of CD98 and GOT2 after tumor challenge (Fig. 3G). Remarkably, IL-7-secreting CD8 + CAR-T cells exhibited greater activity of pS6, CD98, and GOT2 (Fig. 3G), suggesting that IL-7-secreting CD8 + CAR-T cells more efficiently induced activation of mTOR pathway(pS6) and glycolysis process (GOT2) upon antigenic stimulation. CD98, an amino acid transporter, supports lymphocyte clonal expansion, proliferation, and prevention of apoptosis^38^, was rapidly increased in IL-7-secreting CAR-T cells when cultured with tumor cells. However, unlike CD4 + conventional CAR-T cells, CD8 + conventional CAR-T cells showed lower expression level of CD98 when co-cultured with tumor cells, revealing amino acid metabolism mostly active in CD4 + T cells (Fig. 3D,G). We next sought to assess why IL-7-secreting CD8 + CAR-T cells expressed higher level of pS6 and CD98. By analyzing CD57, a marker expressed at terminal differentiation stage, we observed more than 40% CD8 + T cells expressed CD57 in conventional CAR-T cells after co-culture with tumor cells, while about 20% CD8 + T cells were CD57 positive in IL-7-secreting CAR T cells co-cultured with tumor cells (Fig. 3G), demonstrating that conventional CAR signaling over activated CD8 + T cells and most likely induced downregulation of metabolic activity due to leading to terminal differentiation state. Taken together, when compared with consistently high levels of metabolic activity in conventional CAR-T cells, IL-7-secreting CAR-T cells displayed low level of metabolic activity at resting, where energy consumption was low and sufficient to maintain long-term survival. Conversely, in the presence of tumor cells, IL-7-secreting CAR-T cells can rapidly increase the metabolic activity to provide a boost of energy supply for to mount an anti-tumor immune response, rewire a variety of metabolic pathways to meet the metabolic needs and prevent CAR-T cell apoptosis by increasing the expression of CD98 in T cells.

### IL-7 enhances the persistence of CAR-T cells against tumor cells in vitro

**Figure 4:**
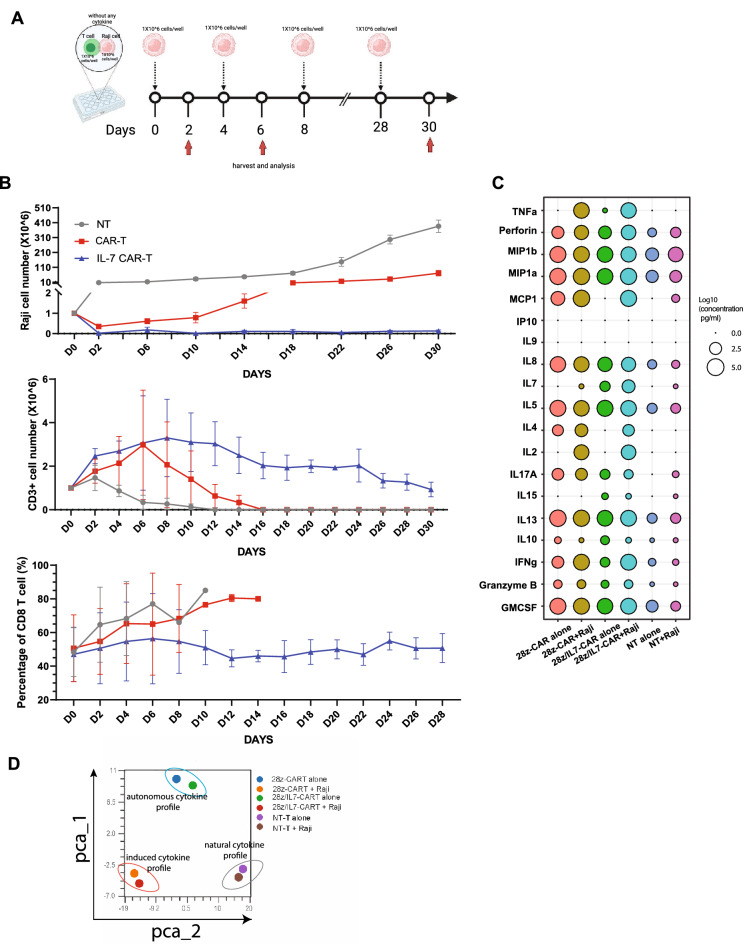
IL-7 enhances the persistence of CAR-T cells in vitro tumor re-challenges. (**A**) A schematic representative of re-challenge experiment to assess CAR-T cell persistence in vitro. (**B**) Plots with lines show the Raji cell number (top), CD3 + T cells (middle) and percentage of CD8 + T cells (bottom) in different days after T cells co-culture with Raji cells. (**C**) Dot plot shows the cytokine production from NT T cells, 28z-CAR-T cells, and 28z/IL-7-CAR-T cells after culturing with or without Raji cells. Dot size indicates the log10 transformed values of cytokine concentration. (**D**) PCA analysis of the cytokine profile of NT T cells, 28z-CAR-T cells, and 28z/IL-7-CAR-T cells.

We next sought to investigate the IL-7-secreting CAR-T cell behavior and efficacy against tumor cells using a long-term tumor challenge model in vitro. 28z/IL-7-CAR-T cells were co-cultured with Raji cells at 1:1 ratio without cytokine support and Raji cells added to the co-culture system every 2 days. As control, 28z-CAR-T cells and NT T cells were treated in parallel similarly (Fig. 4A). To evaluate the anti-tumor efficacy of CAR-T cells, we quantified the tumor cells every 4 days and observed no detectable Raji cells in the culture of 28z/IL-7 CAR-T cells for 30 days, while the conventional CAR-T cells controlled Raji cell growth for the first 10 days, but lost anti-tumor efficacy after 14 days re-challenge (Fig. 4B). A recent report of a CAR-T cell therapy trial with ten years of follow-up reported that CD4 + CAR-T cells were found as the dominant CAR-T cells persisting in two complete remission patients and those CD4 + CAR-T cells exhibited continued functional activation and proliferation^39^. Thus, we measured the absolute count of T cells and the percentage of CD8 + T cells comparison to CD4 + T cells, which could serve as an indirect measure of proliferation . Notably, CD3 + T cell number in 28z-CAR-T cell group peaked on day 6 and we observed a sharp downtrend. However, in 28z/IL-7-CAR-T cell group CD3 + T-cells displayed a longer uptrend, peaked on day 8 plateaued until day 16 (> 2 × 10^6^ cells) and appreciable number of CD3 + T-cells were detectable till day 30. In contrast, the number of CD3 in 28z-CAR-T cell group dropped to zero on day 16 after the peak. By assessing fractions of CD4 + and CD8 + T cells, we concluded that tonic signaling of 28z-CAR-T cells might favor the relative expansion CD8 + T cells and tipped the balance against CD4 + T-cells. However, the overstimulation of CD8 + T cells in the absence of cytokine support could induce exhaustion and T-cell dysfunction. Indeed, we observed that the CD8 + 28z/IL-7-CAR-T cell numbers were maintained at a stable level, but 28z-CAR-T cells were detected as 80% CD8 + T cells at the end of their lifetime in culture but no efficacy to eliminate tumor cells (Fig. 4B, Supplementary Fig. 4). These data revealed that IL-7 promoted CAR-T cell survival, particularly CD4 + T cells that may play a pivotal role in sustaining CD8 + CAR-T cells through secretion of IL-2. To provide another aspect on the anti-tumor response mediated by 28z/IL-7 and 28z CAR-T cells, we also examined the cytokines in supernatants released by NT T cells, 28z-CAR-T cells, and 28z/IL-7-CAR-T cells when cultured with or without tumor cells using ELISA-based cytokine detection assay to assess 19 cytokines. The assessment of overall cytokine secretion profile revealed that both 28z-CAR-T cells and 28z/IL-7-CAR-T cells produced Th1 cytokines, including TNF-α, IFN-γ, as well as Th2 cytokines, including IL-4, IL-5, and IL-13, indicating that CD4 + CAR-T cells were mainly consisted of Th1 and Th2 cells (Fig. 4C). To achieve an overall comparative assessment of cytokine profiles in CAR-T cells, we generated a PCA plot cluster the samples with similar cytokine profiles. Not surprisingly, NT-T cells with or without tumor cell stimulation were clustered together (circled in gray, labeled as natural cytokine profile) indicating that NT T-cells did not respond to tumors with regards to cytokine production. On the other hand, 28z-CAR-T cells and 28z/IL-7-CAR-T cells under tumor cell stimulation were closely situated (bottom left) (circled in red and labeled as induced cytokine profile) , indicating that they respond similarly to challenging with tumor cells. Additionally, resting 28z-CAR-T cells and 28/IL-7-CAR-T cells were clustered together (circled in blue, autonomous cytokine profile) (Fig. 4D). Overall these findings indicate that NT T cells did not respond to tumor cells since they did not express any receptor recognizing a cognate antigen on tumor cells . On the other hand, 28z and 28z/IL-7 CAR-T cells alone showed similar cytokine profiles at baseline, most likely secondary to tonic CAR signaling. Moreover, both 28z and 28z/IL-7 CAR-T cells displayed similar cytokine expression profiles in the presence of tumor cells, which differed from cytokine profiles of resting CAR-T cells. Importantly, IL-7 secretion in the presence and absence of tumor cells, was also detected as a distinct feature of 28z/IL-7 CAR-T cells using the multiplexed cytokine assay. Our findings indicate that 28z/IL-7 CAR-T cells demonstrate comparable cytokine response to 28z CAR-T cells but are equipped with superior persistence capacity.

### IL-7-secreting CAR-T cells are persistent in vivo and associated with superior survival

**Figure 5:**
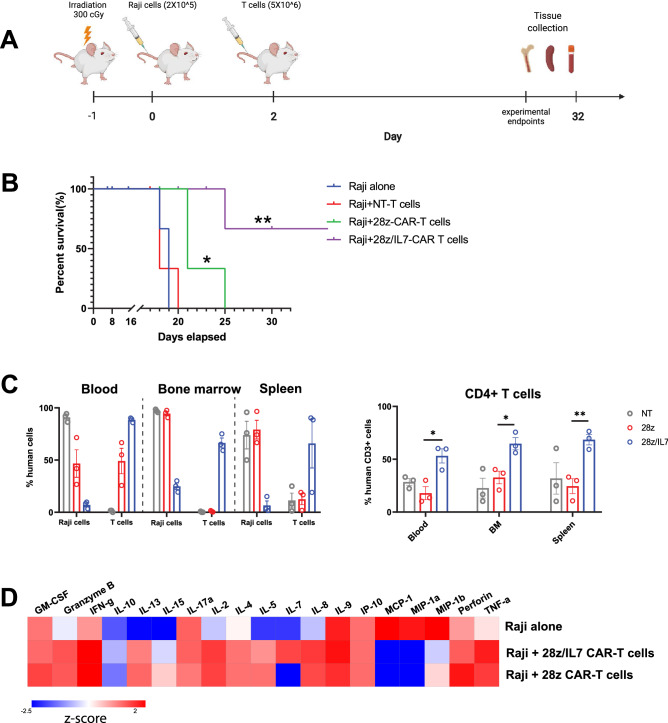
IL-7 secreting CAR T cells are persistent in vivo and associated with superior survival. (**A**) A schematic representative of murine model. (**B**) Kaplan–Meier plots shows the probability of survival for four groups of mice shown in indicated color scheme. Statistical significance is represented by log-rank (Mantel-cox) test *p < 0.05, **p < 0.01. (**C**) Bar plots show the percentage of Raji cells and T cells (left) and CD4 + T cells (right) in peripheral blood and mouse organs (bone marrow and spleen) from three different treatment groups (NT T cells, 28z-CAR-T cells, and 28z/IL7-CAR-T cells) (Error bars indicate median ± SEM, **p < 0.01, ****p < 0.0001, n = 3, unpaired t test). (**D**) Heatmap shows the cytokine production levels in mouse serum from three different groups (Raji alone, Raji + 28z-CAR-T cells, and Raji + 28z/IL7-CAR-T cells).

To test our hypothesis in vivo, we generated a CD19 + xenograft lymphoma murine model using Raji cells. In briefly, Raji cells were injected on day 0 and T cells were injected on day 2. The mice were monitored every day after injection and were sacrificed when mice reached the end point criteria (Fig. 5A). Remarkably, mice treated with either 28z/IL-7 or 28z CAR-T cells had significantly prolonged survival compared to mice treated with NT T-cells (Fig. 5B). Given in vitro our findings that IL-7-secreting CAR-T cells demonstrated enhanced persistence and maintained CD4/CD8 ratio upon antigenic stimulation, we tested CD3 + CAR-T cells in vivo persistence and assessed CD4/CD8 ratio in CD3 + T-cell pool in each organ after mice were sacrificed. We observed that mice treated with NT T cells harbored more than 70% tumor cells in peripheral blood, bone marrow and spleen, and the fraction of CD3 + T cells was less than 10% in peripheral blood, indicating that NT T-cells did not exert any cytotoxicity in vivo. In 28z CAR-T cell group , human CD45 cells detected in peripheral blood were 50% of T-cells while tumor cells were the major cells in bone marrow and spleen when mice were sacrificed. This indicates 28z CAR-T cells had a limited anti-tumor function and failed to control tumor growth. Noteworthy, we assessed the mice organ in the mice treated with 28z /IL-7 CAR-T cells on day 32 after tumor cell injection, CD3 + T cells were the major human cells detected in peripheral blood, bone marrow and spleen (Fig. 5C), supporting the notion that IL-7 promotes CAR-T cell persistence in vivo. Following of discovery if superior CAR-T cell persistence in vivo, we next examined the CD4 + CAR-T cell distribution across different organs in mice. In line with in vitro findings, IL-7-secreting CAR-T cells maintained higher frequencies of CD4 + T cells, compared to CD8 + T-cells, in peripheral blood, bone marrow and spleen. Conversely, in 28z CAR-T cell group, the frequencies of CD4 + T cells were significantly lower in the bone marrow and spleen. Particularly, CD4 + T cells were less than 20% of CD3 + T cells in peripheral blood, suggesting the 28z construct does not support in vivo persistence and expansion of CD4 + T-cells. We next conducted cytokine detection assay on the serum samples collected from mice blood and we detected high levels of Th1 cytokines, including TNF-α and IFN-γ, in both 28z and 28z/IL7 CAR-T groups. On the other hand, we observed lower level of Th2 cytokines, including IL4, IL5, and IL13 in IL-7-secreting CAR-T group than 28z CAR-T group. This suggests that IL-7 could enforce more Th1-prone signature compared to 28z CAR-T group, which could translate to efficient tumor control. Importantly, serum IL-7 levels were detectable in vivo only in 28z/IL-7, which provides further evidence on role of IL-7 in driving T-cell persistence in vivo (Fig. 5D). In summary, ectopic expression of IL-7 enhanced CAR-T cells in vivo persistence by mainly supporting CD4 + T cells , which translated to improved survival in xenograft murine models with CD19 + Raji cells.

## Discussion

The mechanism of primary and acquired resistance has been linked to two distinct categories: 1) T-cell fitness and functional state in the apheresis and infusion products. 2) tumor intrinsic mechanisms of resistance, such as antigen escape and immunosuppressive tumor microenvironment. Due to all those resistance mechanisms, 40–50% of relapsed or refractory diffuse large B-cell lymphoma patients fail to achieve long-term remission^4,40^. To address these challenges related to CAR-T cell therapy in clinic, multiple pre-clinical studies employed diverse strategies of CAR development to overcome or reduce the resistance to CAR-T cell therapy along with second generation CARs including co-stimulatory domain (CD28 and 4–1 BB), and more recently, third generation CARs composed of two different co-stimulatory signaling units in the intracellular regions besides CD3ζ^41^.

Multiple factors that are considered as the key of resistance to CAR-T therapy. One of well-known reason of resistance to CAR-T cell therapy is the inadequate T-cell persistence, which is associated with a higher risk of relapse^42,43^. Thus, the duration of CAR-T cells after infusion are closely associated with clinical outcomes in CAR-T cell therapy^44^. To this end, different subsets of CD4 + /CD8 + T cells were isolated to generate anti-CD19 CAR-T cells based on their differential potential of proliferation, longevity, and functionality CAR-T cells derived from naïve and central memory CD4 + T cells had greater anti-tumor activity and prolonged persistence^45^. T-cell persistence plays a key role in enhancing CAR-T cell therapy efficacy and is linked to T-cell ability for self-renewal and proliferation, functional attributes enriched in CD4 + naïve and central memory T cells. We armored 28z CAR-T cells (CD28 co-stimulator) with IL-7 to enhance cell proliferation and effector functions, which could improve tumor cell elimination. As expected, CAR-mediated cytotoxicity of T cells induced tumor cell killing. Moreover, less differentiated phenotype was an observed only in IL-7-secreting CAR-T cells, with retained CCR7 expression on CD4 + T cells after expansion in vitro. IL-7 prevented CAR-T cells from exhaustion, particularly in CD4 + T cells and maintained CD4 + T-cells in a less-differentiated state.

As for T-cell metabolic fitness, T cells inherit different metabolic pathways based on their differentiation stage and memory status. With homeostatic cytokines like IL-7, naïve T cells can achieve long lifespan^46^, and remain viable by the usage of OXPHOS at the resting stage. However, upon differentiation into effector T cells, the use of glucose is required for the proliferation and cytotoxic activity^47^. In line with the fundamentals of T-cell metabolism to exert a potent immune response, we investigated the metabolic profiles of 28z and 28z/IL-7 CAR-T cells in resting and after antigen exposure. Significantly higher level of metabolic activity was observed in 28z CAR-T cells at resting stage, which may due to tonic signaling from CAR molecule. In turn, robust metabolic activation was occurred in 28z/IL-7 CAR-T cells in the presence of tumor cells. Together, our results suggest that IL-7 plays a role in maintaining CAR-T cells in a less differentiated state and minimizing energy consumption/metabolic activity at the resting stage, without inhibiting the metabolic activation when CAR-T cells were exposed to antigenic stimulation. To assess the utility of this strategy for clinical translation, we treated B-cell lymphoma xenograft mice with 28z and 28z/IL-7 CAR-T cells were persistent in vivo, rapidly diminished the tumor cell burden, and achieved superior survival , indicating the mechanism underlying the functional and proliferative advantage observed in vitro is mainly driven by IL-7.

In this study, we did not assess the toxicity that could be associated with CAR-T cells secreting IL-7. However, we did not see any sign of toxicity in mice treated with 28z/IL-7 CAR-T cells. Moreover, we observed that the cytokine profiles of 28z and 28z/IL-7 CAR-T cells in vitro and in vivo displayed similarity per 19 cytokines that we assessed using multiplexed cytokine assay. Thus, we assume that the 28z/IL-7 CAR-T cells may not be associated with more toxicity and adverse effects compared to 28z CAR-T cells. Moreover, previous studies showed that IL-7 was well tolerated in human or only mild symptoms were observed^48,49^.

In summary, our data showed the efficacy and utility of a novel CAR design strategy to overcome CAR-T cell exhaustion and dysfunction, providing a clinical rationale of CAR signaling modulation and metabolism reprogramming through using IL-7 in CAR construct to enhance CAR-T cell persistence and induce durable remission upon CAR-T cell therapy.

## Materials and methods

### CAR 19 cloning and lentivirus production

DNA fragments encoding the FMC63 (anti-CD19) ^50^ were synthesized (Genescript) and subcloned into pLJM1(Addgene) in frame with the sequence encoding the IgG1 hinge, the transmembrane domain of CD28 with intracellular domains of CD3z and/or IL-7 under the EF1a promoter, as shown in Fig. 1A. To produce lentiviral particles for transduction into T cells, HEK293T cells were transfected with the vector carrying CAR19 together with the packaging plasmids psPAX2 and pMD2.G (Addgene). Viral supernatants were collected after 48 h of incubation.

The study was approved by the Review Board of Shanghai Ruijin Hospital and all experimental protocols were approved by the Review Board of Shanghai Ruijin Hospital. Informed consent was obtained from all the participants.

### T-cell transduction and culture conditions

T cells were isolated from healthy donor peripheral blood mononuclear cells (PBMCs) by a pan-T cell selection kit (Miltenyi) following the manufacturer’s instructions. Cells were then activated, expanded for 48 h using CD3/CD28 beads (Dynabeads, Gibco), transduced 48 h later with the lentivirus, and incubated overnight in RPMI 1640 with 10% fetal bovine serum (FBS) and IL-2 (300 IU/ml). CAR was detected using anti-human IgG antibody (ThermoFisher, Cat #. 31,165).

### Cytotoxic potency assay

The cytotoxic potency of CAR-T cells was assessed by bioluminescence (BLI) based cytotoxicity assay^51^. Briefly, luciferase-transduced Raji cells were co-incubated with T cells for 18 h at an serially diluted effector-to-target (E:T) ratios of 4:1, 2:1, 1:1, 1:2, 1:4 and 1:8. BLI was then measured with luminometer (IVIS Lumina X5) as relative light units (RLU). Percent lysis was calculated from the triplicate wells as % lysis = (spontaneous death RLU—test RLU)/(spontaneous death RLU—maximal killing RLU) × 100%, where maximal killing was induced by incubation in a 1% Triton X-100 solution.

### CAR-T cells re-challenge experiment

NT-T cells , 28z-CAR-T cells and 28z/IL-7 CAR-T cells were co-cultured with Raji cells at 1:1 ratio (1 × 10^6 cells) in 24 well plate without any cytokine in the RPMI 1640 medium ( Thermo Fisher, CAT. 11,875,093) with 10% fetal bovine serum ( Thermo Fisher, cat #: 10,082) in 37ºC , 5% CO_2_ incubator, and re-challenged with 1 × 10^6 cells Raji cells/well every 4 days until day 28. Analysis of tumor cells and T cells number, as well as assessment of CD4/CD8 ratio were performed every other day.

### Cytokine profiling

Two different CAR engineered T cells from healthy donors were cultured with Raji cells at 1:1 ratio for 18 h. After incubation, supernatants were collected and loaded to CodePlex chip (IsoPlexis) that allowed the assessment of 22 human cytokines per manufacturer’s instructions. Chips were read in IsoLight instrument and data was analyzed in Isospeak software.

### Cell staining and data acquisition

T cells with different treatments were stained with CyTOF/flow antibody mixture against surface/intracellular markers at room temperature, and after washing step, stored overnight in 500 µl of 1.6% paraformaldehyde (PFA) (EMD Biosciences)/PBS with 125 nM iridium nucleic acid intercalator (Fluidigm)^32^. Cells were washed twice with a cell staining buffer and twice with dH_2_O before acquisition. Data were acquired on a Helios mass cytometer (Fluidigm). Fluorescence cytometry data were acquired on BD LSRFortessa X-20 (BD Biosciences).

### Data analysis

#### Exhaustion score and metabolic activity score

T-cell exhaustion score was calculated by the mean expression of four canonical markers of T cells exhaustion: PD-1, CTLA-4, TIM-3, and LAG3, as previous study described^52^. Using the same method, we calculated the metabolic activity score with the mean expression of 17 markers related to metabolic activation pathways: ASCT2, C-Myc, Glut-1, OPA-1, GLS, CD98, GOT2, CD39, IDH-1, GAPDH, Cytochrome C, MCT-4, CTLA-4, CPT1, pS6, pNRF2, DRP1, and IRF4.

#### Diversity index calculations

Diversity was computed on the basis of the Boolean expression of 32 markers related to T cell differentiation, exhaustion, function and activation : 2B4, CD25, CCR2, CCR4, CCR6, CCR7, CD26, CD27, CD28, CD36, CD45RA, CD49F, CD57, CD69, CD127, CD137, CD160, CTLA-4, CXCR3, EOMES, Granzyme A, HLA-DR, IDU, KI67, KLRG1, LAG3, PD-1, PERFORIN, TBET, TIGIT, TIM-3 and TRAIL, using the inverse Simpson Index as previously described^32^.

#### Dimension reduction

Omiq software was utilized for dimension reduction and visualization of the CyTOF data in two dimensions. Pooled NT T cells, 28z-CAR-T cells and 28z/IL-7 CAR-T cells were subjected to UMAP dimension reduction. To assess the phenotypic profiles, clusters were generated using Omiq and expression of each marker was shown using UMAP plots.

#### Pseudo-time inference

Diffusion was utilized to perform trajectory analysis and pseudo-time inference for NT cells, 28z-CAR-T cells and 28z/IL-7-CAR-T cells. Expression values were transformed by Arcsinh and cells were subjected to UMAP dimension reduction to project the data in two dimensions. Diffusion maps were performed to identify the major trend in the data as a part of trajectory analysis and three informative diffusion components were selected (CCR7, CD45RA and CD57) based on plotting the diffusion components vs algorithm-generated eigenvalue for trajectory analysis. The data was plotted in two-dimensions using UMAP plot and projected the pseudo-time values on UMAP plots to infer cellular relatedness along pseudo-time.

#### Xenograft model

All the animal experiments were performed in adherence to the laboratory animal welfare and ethics committee, which was approved by the Ethics Committee of Ruijin Hospital. And all methods are reported in accordance with ARRIVE guidelines. NOD-scid gamma (NSG) mice (six to eight weeks old female, the Jackson Laboratory) were irradiated with 300 cGy one day prior to intravenous injection with FFLuc-labeled Raji (1 × 10^5^/mouse) cells on day 0. Expanded NT or CAR-T cells (5 × 10^6^/mouse) were injected through the tail vein on day 2. Mice were sacrificed when either experimental or humane endpoints were reached. Murine spleen, blood and bone marrow were harvested and cryopreserved for later analysis by flow cytometry.

#### Statistical analysis

Statistical analyses were performed using Prism version 7.0 (GraphPad Software Inc.). The statistical differences between matched groups were compared using a paired T test using GraphPad Software. Mann–Whitney U test was used to compare unpaired groups and Spearman rank correlation was used to assess correlation. (Statistical significance: *p < 0.05; **p < 0.01; ***p < 0.001; and ****p < 0.0001).

## Supplementary Information

Supplementary Information.

## Data Availability

The datasets generated and analyzed during the current study are available in the OSF repository, https://osf.io/fxst8/.

## References

[1] Mullard A (2021). FDA approves fourth CAR-T cell therapy. Nat. Rev. Drug Discov..

[2] Neelapu SS (2017). Axicabtagene ciloleucel CAR T-cell therapy in refractory large B-cell lymphoma. N. Engl. J. Med..

[3] Munshi NC (2021). Idecabtagene vicleucel in relapsed and refractory multiple myeloma. N. Engl. J. Med..

[4] Park JH (2018). Long-term follow-up of CD19 CAR therapy in acute lymphoblastic leukemia. N. Engl. J. Med..

[5] Porter DL, Levine BL, Kalos M, Bagg A, June CH (2011). Chimeric antigen receptor-modified T cells in chronic lymphoid leukemia. N. Engl. J. Med..

[6] Schuster SJ (2019). Tisagenlecleucel in adult relapsed or refractory diffuse large B-cell lymphoma. N. Engl. J. Med..

[7] Fraietta JA (2018). Determinants of response and resistance to CD19 chimeric antigen receptor (CAR) T cell therapy of chronic lymphocytic leukemia. Nat. Med..

[8] Zhang G (2014). Anti-melanoma activity of T cells redirected with a TCR-like chimeric antigen receptor. Sci. Rep..

[9] Oren R (2014). Functional comparison of engineered T cells carrying a native TCR versus TCR-like antibody-based chimeric antigen receptors indicates affinity/avidity thresholds. J. Immunol..

[10] Rafiq S (2017). Optimized T-cell receptor-mimic chimeric antigen receptor T cells directed toward the intracellular Wilms Tumor 1 antigen. Leukemia.

[11] Mata M (2017). Inducible activation of MyD88 and CD40 in CAR T cells results in controllable and potent antitumor activity in preclinical solid tumor models. Cancer Discov..

[12] Guedan S (2014). ICOS-based chimeric antigen receptors program bipolar TH17/TH1 cells. Blood.

[13] Dai H (2020). Bispecific CAR-T cells targeting both CD19 and CD22 for therapy of adults with relapsed or refractory B cell acute lymphoblastic leukemia. J. Hematol. Oncol..

[14] Fernández de Larrea C (2020). Defining an optimal dual-targeted CAR T-cell therapy approach simultaneously targeting bcma and gprc5d to prevent bcma escape-driven relapse in multiple myelomaaddressing bcma escape with dual-targeted car t-cell therapy. Blood Cancer Discov..

[15] Pegram HJ (2012). Tumor-targeted T cells modified to secrete IL-12 eradicate systemic tumors without need for prior conditioning. Blood.

[16] Hoyos V (2010). Engineering CD19-specific T lymphocytes with interleukin-15 and a suicide gene to enhance their anti-lymphoma/leukemia effects and safety. Leukemia.

[17] Adachi K (2018). IL-7 and CCL19 expression in CAR-T cells improves immune cell infiltration and CAR-T cell survival in the tumor. Nat. Biotechnol..

[18] Markley JC, Sadelain M (2010). IL-7 and IL-21 are superior to IL-2 and IL-15 in promoting human T cell-mediated rejection of systemic lymphoma in immunodeficient mice. Blood.

[19] Brocker T, Karjalainen K (1995). Signals through T cell receptor-zeta chain alone are insufficient to prime resting T lymphocytes. J. Exp. Med..

[20] Allison KE, Coomber BL, Bridle BW (2017). Metabolic reprogramming in the tumour microenvironment: A hallmark shared by cancer cells and T lymphocytes. Immunology.

[21] Pearce EL (2009). Enhancing CD8 T-cell memory by modulating fatty acid metabolism. Nature.

[22] Bradley LM, Haynes L, Swain SL (2005). IL-7: Maintaining T-cell memory and achieving homeostasis. Trends Immunol..

[23] ElKassar N, Gress RE (2010). An overview of IL-7 biology and its use in immunotherapy. J. Immunotoxicol..

[24] Mu-Mosley H (2022). Transgenic expression of IL15 retains CD123-redirected T Cells in a less differentiated state resulting in improved anti-AML activity in autologous AML PDX models. Front. Immunol..

[25] Cieri N (2013). IL-7 and IL-15 instruct the generation of human memory stem T cells from naive precursors. Blood.

[26] Tian Y (2017). Unique phenotypes and clonal expansions of human CD4 effector memory T cells re-expressing CD45RA. Nat. Commun..

[27] Okoye AA (2015). Effect of IL-7 therapy on naive and memory T cell homeostasis in aged rhesus macaques. J. Immunol..

[28] Gattinoni L, Klebanoff CA, Restifo NP (2012). Paths to stemness: Building the ultimate antitumour T cell. Nat. Rev. Cancer.

[29] Gattinoni L (2011). A human memory T cell subset with stem cell-like properties. Nat. Med..

[30] Behbehani GK, Bendall SC, Clutter MR, Fantl WJ, Nolan GP (2012). Single-cell mass cytometry adapted to measurements of the cell cycle. Cytometry A.

[31] Long AH (2015). 4–1BB costimulation ameliorates T cell exhaustion induced by tonic signaling of chimeric antigen receptors. Nat. Med..

[32] Li L (2019). A novel immature natural killer cell subpopulation predicts relapse after cord blood transplantation. Blood Adv..

[33] Qi Q (2014). Diversity and clonal selection in the human T-cell repertoire. Proc. Natl. Acad. Sci. U.S.A..

[34] Calderon H, Mamonkin M, Guedan S (2020). Analysis of CAR-mediated tonic signaling. Methods Mol. Biol..

[35] Buck MD (2016). Mitochondrial dynamics controls T cell fate through metabolic programming. Cell.

[36] Corrado M (2021). Deletion of the mitochondria-shaping protein Opa1 during early thymocyte maturation impacts mature memory T cell metabolism. Cell Death Differ..

[37] Angela M (2016). Fatty acid metabolic reprogramming via mTOR-mediated inductions of PPARgamma directs early activation of T cells. Nat. Commun..

[38] Cantor JM, Ginsberg MH (2012). CD98 at the crossroads of adaptive immunity and cancer. J. Cell Sci..

[39] Melenhorst JJ (2022). Decade-long leukaemia remissions with persistence of CD4+ CAR T cells. Nature.

[40] Roschewski M, Longo DL, Wilson WH (2021). CAR T-cell therapy for large B-cell lymphoma—Who, when, and how?. N. Engl. J. Med..

[41] Guedan S, Calderon H, Posey AD, Maus MV (2019). Engineering and design of chimeric antigen receptors. Mol. Ther. Methods Clin. Dev..

[42] Turtle CJ (2016). CD19 CAR-T cells of defined CD4+:CD8+ composition in adult B cell ALL patients. J. Clin. Investig..

[43] Gardner RA (2017). Intent-to-treat leukemia remission by CD19 CAR T cells of defined formulation and dose in children and young adults. Blood.

[44] Maude SL (2014). Chimeric antigen receptor T cells for sustained remissions in leukemia. N. Engl. J. Med..

[45] Sommermeyer D (2016). Chimeric antigen receptor-modified T cells derived from defined CD8+ and CD4+ subsets confer superior antitumor reactivity in vivo. Leukemia.

[46] Nasi M (2006). Thymic output and functionality of the IL-7/IL-7 receptor system in centenarians: Implications for the neolymphogenesis at the limit of human life. Aging Cell.

[47] Rangel Rivera GO (2021). Fundamentals of T cell metabolism and strategies to enhance cancer immunotherapy. Front. Immunol..

[48] Sportes C (2010). Phase I study of recombinant human interleukin-7 administration in subjects with refractory malignancy. Clin. Cancer Res..

[49] Kim JH, Lee KJ, Lee SW (2021). Cancer immunotherapy with T-cell targeting cytokines: IL-2 and IL-7. BMB Rep..

[50] Zola H (1991). Preparation and characterization of a chimeric CD19 monoclonal antibody. Immunol. Cell Biol..

[51] Karimi MA (2014). Measuring cytotoxicity by bioluminescence imaging outperforms the standard chromium-51 release assay. PLoS ONE.

[52] Wilk AJ (2020). A single-cell atlas of the peripheral immune response in patients with severe COVID-19. Nat. Med..

