# Supplementary material for: Transgenic expression of IL-7 regulates CAR-T cell metabolism and enhances in vivo persistence against tumor cells
Source: Sci Rep. 2022 Jul 22;12:12506. doi: 10.1038/s41598-022-16616-2 (PMC9307822; doi:10.1038/s41598-022-16616-2)
Supplement: Supplementary file 1 — Supplementary Information. [file 41598_2022_16616_MOESM1_ESM.pdf]

Graph Abstract

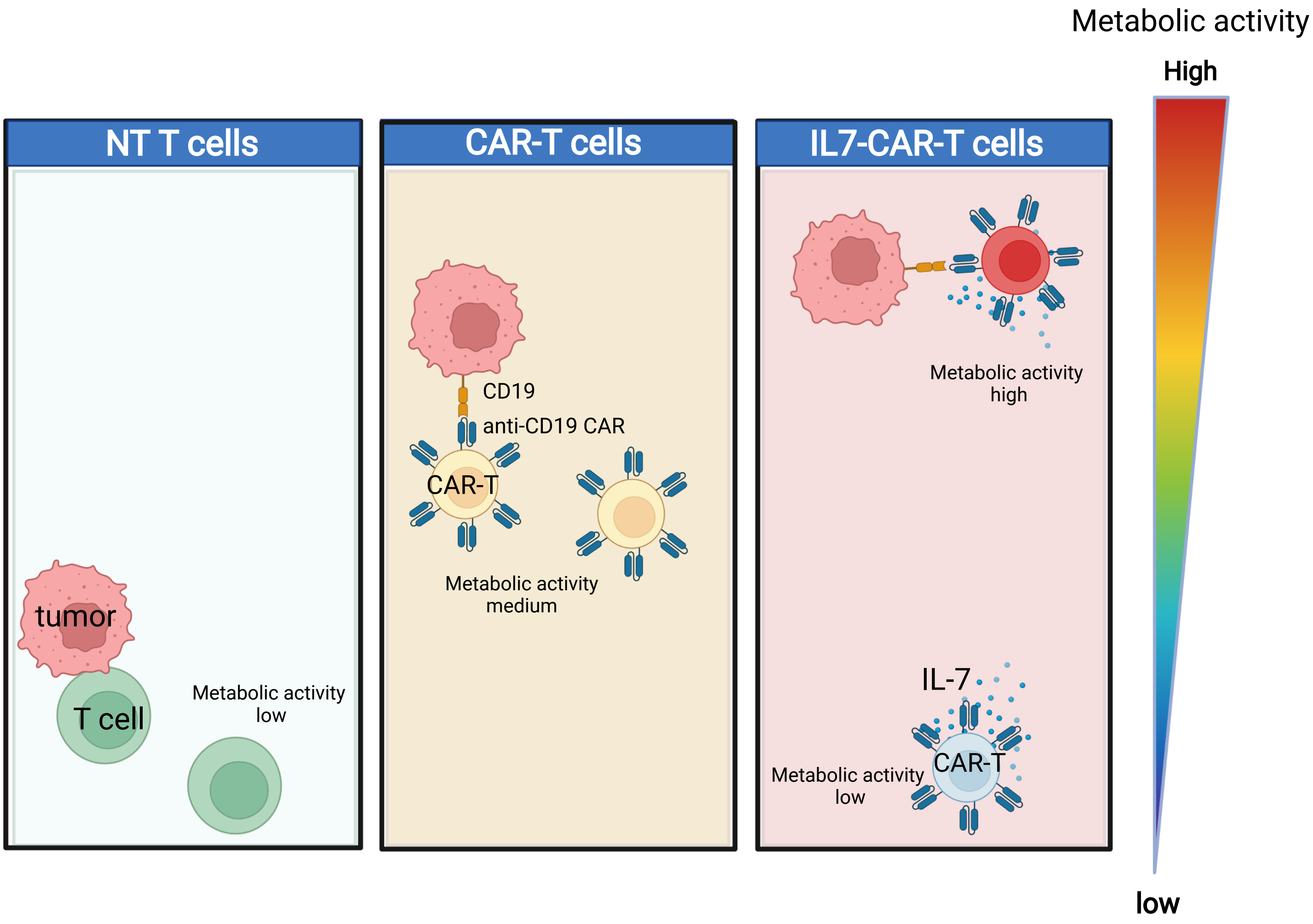

Supplementary Table S1. List of antibodies used for 35-parameter mass cytometry panel to characterize the T cells phenotype.

Tag	Antibody	Clone	Source
113In	CD8	OKT8	Thermo Fisher
115In	CD3	HIT3a	Biolegend
127I	IDU	32D8.D9	Thermo Fisher
139La	CD36	5-271	BIolegend
141Pr	CCR6	G034E3	BIolegend
144Nd	CD26	M-A261	BD Biosciences
145Nd	CD39	A1	BIolegend
148Nd	CCR2	K036C2	BIolegend
149Sm	CCR4	L291H4	BIolegend
150Nd	CD28	CD28.2	BIolegend
151Eu	Tbet	4B10	Biolengend
152Sm	TIGIT	MBSA43	Thermo Fisher
153Eu	CD45RA	HI100	Biolengend
154Sm	Granzyme A	CB9	Biolegend
155Gd	CCR7	G043H7	Biolegend
156Gd	TIM3	F38-2E2	Biolegend
158Gd	2B4	2-69	Biolegend
159Tb	FOXP3	PCH101	Fluidigm
160Gd	CD19	HIB19	Biolegend
161Dy	CXCR3	G025H7	Biolegend
162Dy	Eomes	WD1928	Thermo Fisher
163Dy	CD137	QA18A49	Biolegend
164Dy	CTLA-4	BNI3	Biolegend
165Ho	CD25	2A3	BD Biosciences
166Er	LAG3	11C3C65	Biolegend
167Er	Perforin	B-D48	Abcam
168Er	CD160	BY55	Biolegend
169Tm	CD69	FN50	Biolegend
170Er	CAR	Polyclonal	Jackson immune research
171Yb	CD4	RM4-5	Biolegend
172Yb	KLRG1	13F12F2	Thermo Fisher
173Yb	PD1	EH12.2H7	Biolegend
174Yb	TRAIL	MD5-1	Biolegend
176Yb	KI67	KI67	Biolegend
194Pt	CD57	HCD57	Biolegend

Supplementary Table S2. List of antobodies used for 32-parameter mass cytometry panel to characterize the T cells metabolic profile.

Tag	Antibody	Clone	Source
113In	CD8	OKT8	Thermo Fisher
115In	CD3	HIT3a	Biolegend
127I	IDU	32D8.D9	Thermo Fisher
139La	CD36	5-271	BIolegend
141Pr	GAPDH	FF26A/F9	BIolegend
142Nd	CD98	MEM-108	BIolegend
144Nd	GOT2	PA5-35374	Thermo Fisher
145Nd	CD39	A1	BIolegend
146Nd	IDH-1	PA5-14358	Thermo Fisher
148Nd	CD4	RM4-5	Biolegend
149Sm	Cytochrome C	37BA11	Thermo Fisher
150Nd	Granzyme K	CB9	BD Biosciences
152Sm	MCT4	22787-1-AP	Thermo Fisher
153Eu	CD45RA	HI100	Biolengend
154Sm	KI67	KI67	Biolegend
155Gd	CCR7	G043H7	Biolegend
156Gd	TIM3	F38-2E2	Biolegend
160Gd	CD19	HIB19	Biolegend
161Dy	CXCR3	G025H7	Biolegend
162Dy	ASCT2	D7C12	Cell signaling
163Dy	cmyc	9E10	Thermo Fisher
164Dy	CTLA-4	BNI3	Biolegend
166Er	Glut-1	SA0377	Thermo Fisher
167Er	OPA1	JM81-35	Thermo Fisher
168Er	GLS	PA5-35365	Thomo Fisher
169Tm	CD69	FN50	Biolegend
170Er	CAR	Polyclonal	Jackson immune research
171Yb	Cpt1	15184-1-AP	Thermo Fisher
172Yb	PS6	D57.2.2E	Cell signaling
173Yb	pNRF2	EP1809Y	ABCAM
174Yb	DRP1	EPR19274	ABCAM
176Yb	IRF4	3E4	Thermo Fisher

Supplemental figure 1

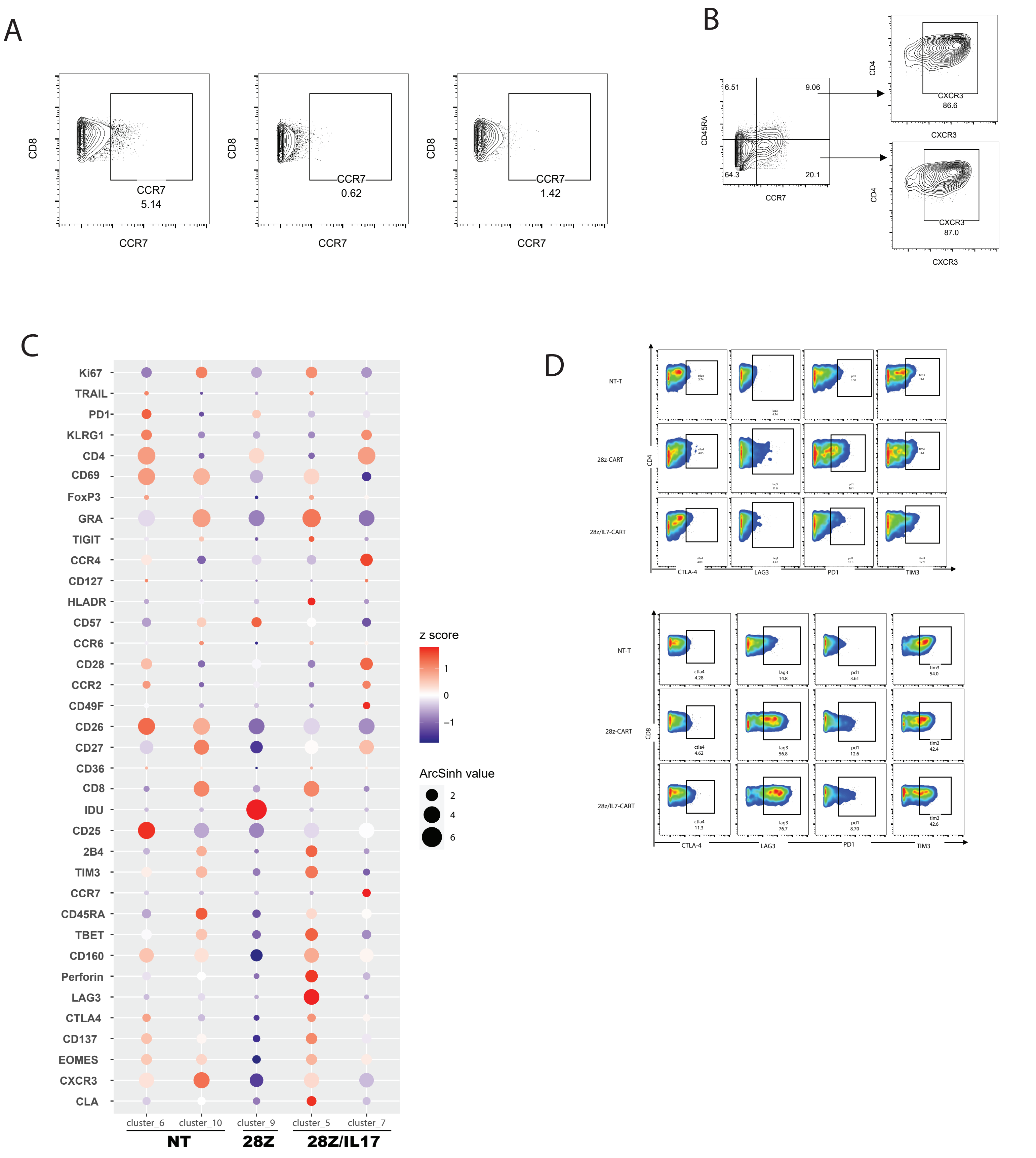

Supplemental figure 1. **IL-7 secreting CAR-T cells displayed less differentiation phenotype**. (**A**) FACS plots show the expression of CCR7 in CD8+ NT T cells, 28z-CAR T cells and 28z/IL7-CAR T cells. (**B**) FACS plots show the expression of CXCR3 in CD45+CCR7+ and CD45-CCR7+ population in CD4+ T cells . (**C**) Dot plot shows the expression of proteomic markers in cluster 6 and 10 of NT T cells, cluster9 of 28z-CAR T cells and cluster 5 and 7 of 28z/IL7-CAR T cells. (**D**) Frequency of native, Tcm, Tem and Temra T cells in + NT T cells, 28z-CAR T cells and 28z/IL7-CAR T cells. (D) FCS plots show the expression of CLT-4, LAG3,PD1 and TIM3 on CD4 (top) and CD8 (down) T cells

Supplemental fiugre 2

A

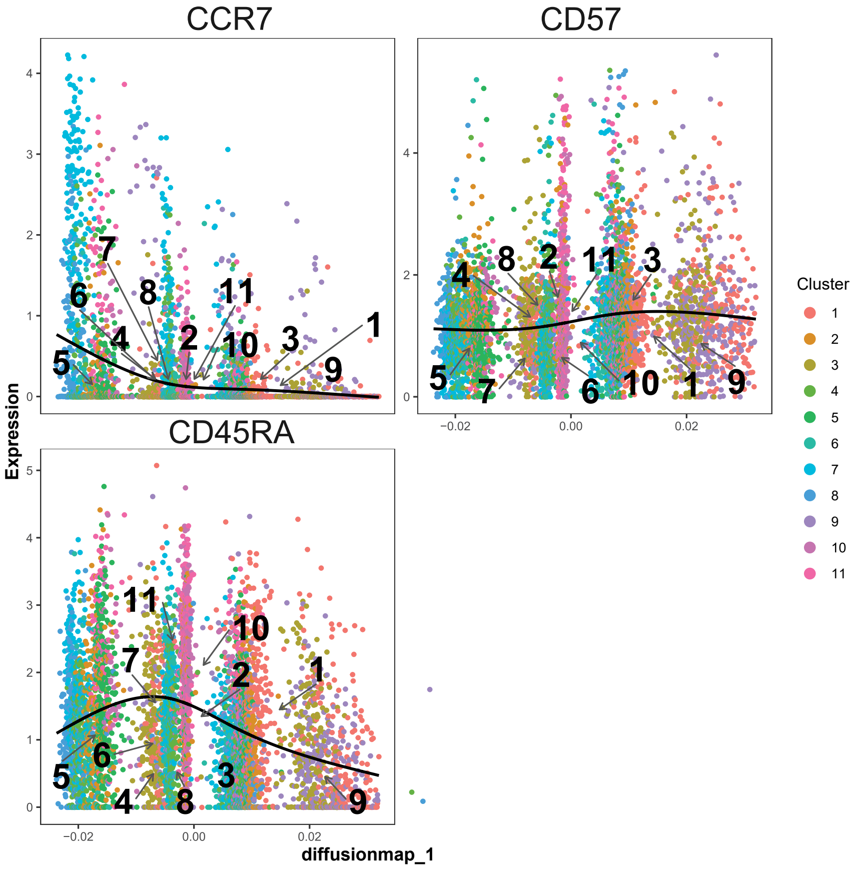

B

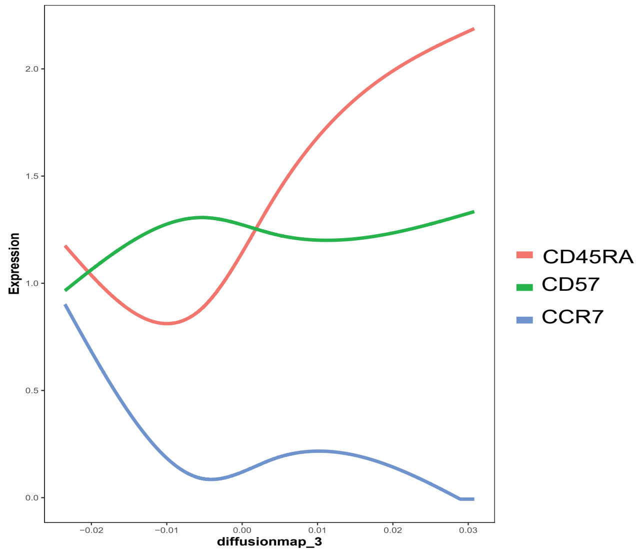

C

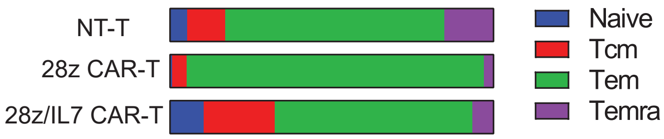

Supplemental figure 2. Trajectory analysis of CAR engineered T cells. (**A**) Diffusion map shows the expression of CCR7, CD57 and CD45RA in 11 clusters and the line shows the median of expression for each cluster. (**B**) Trend line shows the expression of CCR7, CD57 and CD45RA according to the diffusion map ranking. (**C**) Frequency of native, Tcm, Tem and Temra T cells in + NT T cells, 28z-CAR T cells and 28z/IL7-CAR T cells.

Supplemtenal Figure 3

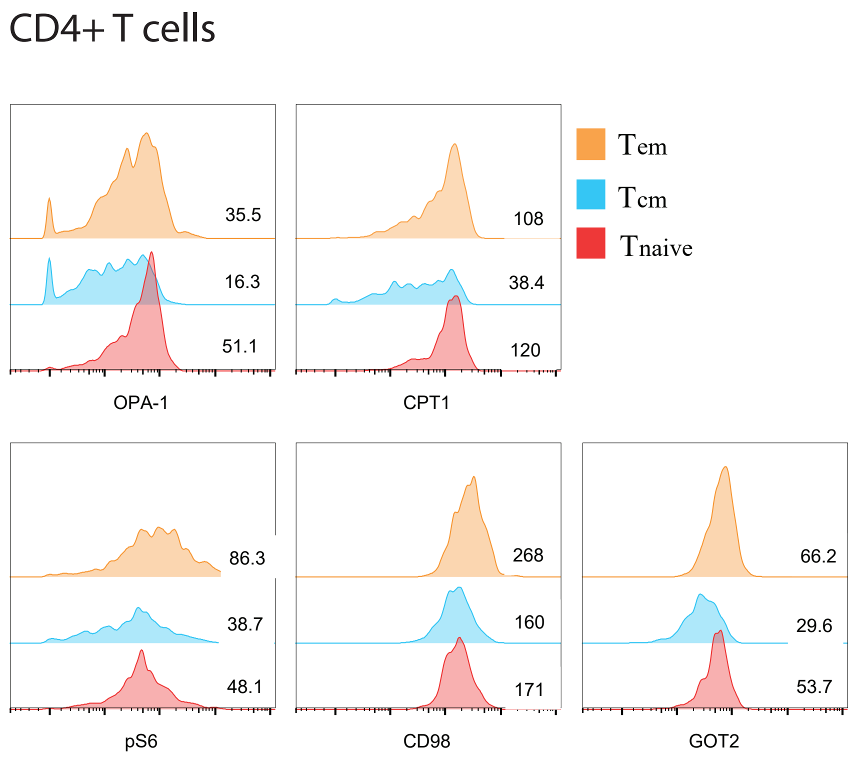

Supplemental figure 3. Histogram plots show the expression of OPA1, CPT1, pS6, CD98 and GOT2 of Tem, Tcm and Tnaive CD4+ T cells.

Supplementary Figure 4

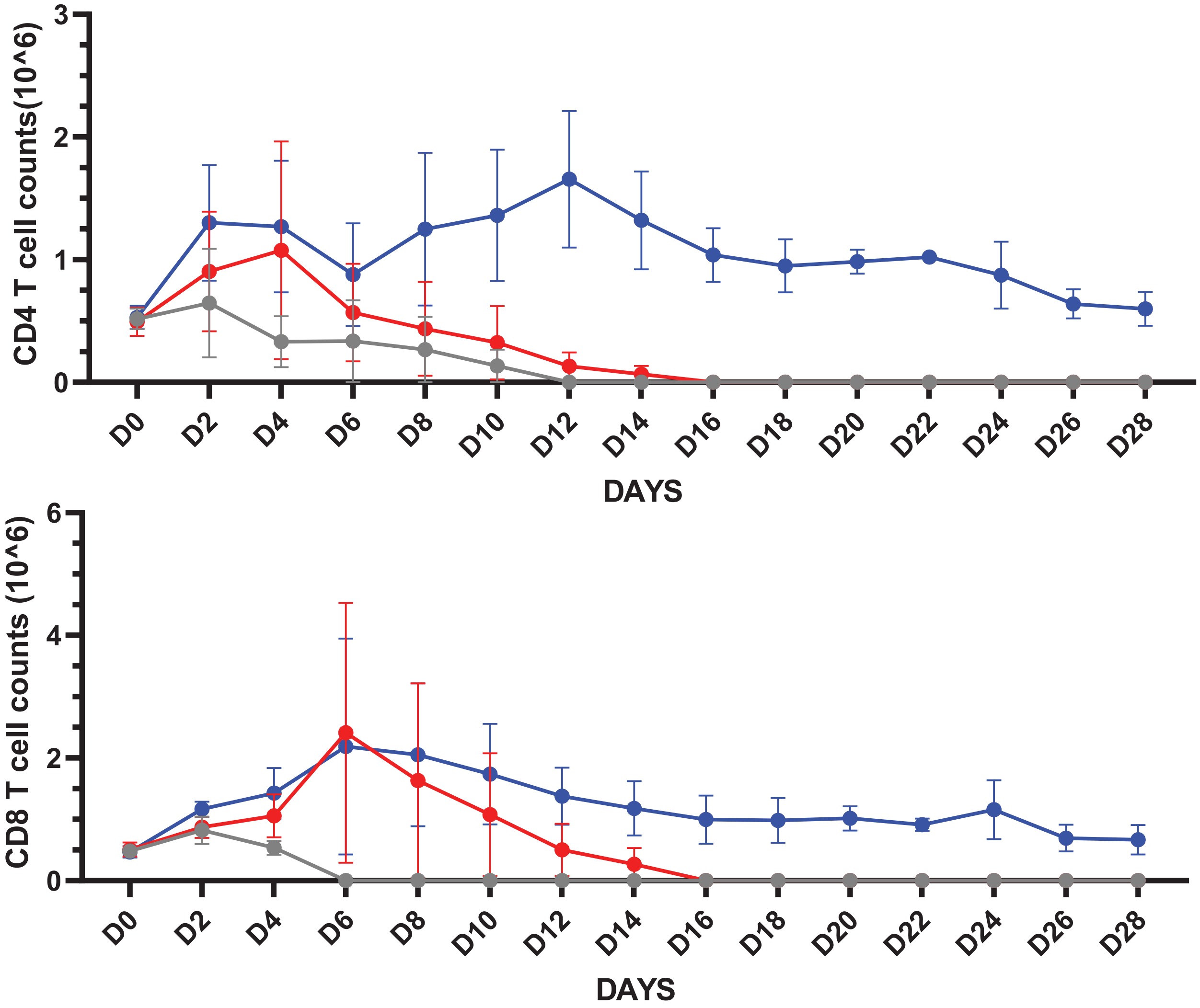

Supplementary Figure 4, Plots with lines show the CD4+ and CD8+ T cell number in different time points after T cells co-culture with Raji cells
